# Quantitative evaluation of traditional Chinese medicine development policy: A PMC index model approach

**DOI:** 10.3389/fpubh.2022.1041528

**Published:** 2022-11-18

**Authors:** Ciran Yang, Shicheng Yin, Dan Cui, Zongfu Mao, Yu Sun, Changli Jia, Shuhan An, Yuxin Wu, Xue Li, Yixin Du, Qiuling Zhao, Rui Wang, Yunxu Liu, Junming Ren, Xue He

**Affiliations:** ^1^Department of Global Health, School of Public Health, Wuhan University, Wuhan, China; ^2^Global Health Institute, Wuhan University, Wuhan, China; ^3^Department of Nursing, School of Nursing, Wuhan University, Wuhan, China

**Keywords:** traditional Chinese medicine, PMC index model, health policy design, health policy structure, quantitative policy evaluation

## Abstract

**Background:**

Traditional Chinese medicine development policies (TCMDPs) are essential in improving the sustainable development of TCM undertakings, of which transmissions of policy information are closely related to the actual policy effectiveness. However, the inherent components of TCMDPs had not been explored from the structural dimension of policy design.

**Methods:**

Based on the policy modeling consistency (PMC) index model, we constructed a comprehensive evaluation system, including ten first-level and 40 second-level indicators, and focused on the TCMDPs released by the Chinese central government in the past 42 years (1980–2022) to conduct multi-dimensional inspections to TCMDPs by analyzing the overall policy quality, individual scoring performance, and indicators distribution characteristics.

**Results:**

This study pointed out that four policies were rated as “perfect,” 35 were rated as “superb,” 50 were rated as “excellent,” 28 were rated as “good,” and four were rated as “acceptable,” with total mean values of the PMC index being 7.530 ± 0.835. Although most TCMDPs had appropriate policy structure and consistency, the potential weaknesses in the design of TCMDPs also needed our attention through careful checks on the outlier policy samples. Besides, the existing TCMDPs had room for improvement regarding policy areas, guarantees and incentives, objects included, and issuing agencies.

**Conclusions:**

We emphasized that the policy evaluation method used in this current study, the PMC index model, is scarce in the TCMDPs. These findings are helpful for fully understanding the strengths and weaknesses of TCMDPs and provide theoretical references for further studies optimizing TCMDPs.

## Introduction

Traditional Chinese medicine (TCM) is a precious creation of the Chinese nation and a treasure from ancient Chinese science, which plays a unique and lasting role in the long history of the Chinese nation's proliferation and inheritance of Chinese culture (1). The essential aspect of TCM, its holistic perspective and personalized therapeutics, is an integral knowledge system for disease prevention (2) and encourages people to focus on and participate in self-care activities (3, 4). In recent years, with the improvement of people's living conditions and the expectations for better health status, people have paid more attention to the role of TCM in disease prevention, health care, and physical rehabilitation (4–6). The public's demand for TCM services is also increasing alongside these developments and changes (7). Especially in response to the COVID-19 epidemic, TCM had been applied in the treatment of confirmed cases throughout the entire process, which had moved the time point of medical intervention forward, effectively prevented the worsening symptoms of the confirmed cases, and made the intervention effects of TCM been fully proved and widely recognized (8–13). It is not difficult to see that, in the current, TCM undertakings are ushering in an extraordinary historical opportunity. For a long time, however, due to the interferences and obstacles of various factors such as its research limitations, traditional atmosphere, misleading information, and lack of professional talents, the inheritance, innovation, and continuous development of TCM still face many difficulties and challenges (14, 15).

The inheritance and innovation of TCM, as a necessary part of the medical and health system construction in China and an important systematic project integrating traditional culture and modern science (16), are inseparable from designing a series of policies and improving institutional mechanisms. Historically, the Chinese government has generally played an active role in promoting the development of TCM undertakings (17). Especially in recent years, the Chinese government has paid unprecedented attention to TCM development. Looking back on the history of policy formulation in the past 40 years, the Chinese government has issued a large number of TCM development policies (TCMDPs), from strategic plannings to concrete measures, which has realistically formed a set of relatively applicable policy system (18), providing political solid guarantees and policy supports for the continuous promotion of the inheritance and innovation of the TCM undertakings. As the number of policies increases and the policy system becomes complex, new troubles also arise: What were the TCMDPs issued at the national level, and what was the long-term trend in the number of policy releases? What were the specific manifestations of these policies' overall quality and individual differences? How do we identify the advantages and disadvantages in policy design and give targeted optimization suggestions? Such questions depend on scholars in the field of public health to give particular attention and solutions.

This study, therefore, set out to focus on the TCMDPs released by the Chinese central government in the past 42 years (1980–2022) and build a scientific and reasonable evaluation indicator system based on the policy modeling consistency (PMC) index model to conduct multi-dimensional inspections to TCMDPs by analyzing the overall policy quality, individual scoring performance, and indicators distribution characteristics, which allows us to give more precise answers to the questions mentioned above and provide some supports for the subsequent improvement of TCMDPs.

## Literature review

### TCMDPs research

Due to the continuous efforts of the Chinese government in formulating and implementing TCMDPs for a long time, TCM has been well integrated into the health care system and has gradually played an essential role in improving public health in China (19). However, after reviewing the literature, we roughly found that although the number of previous studies about TCMDPs was small and the Chinese literature was more abundantly documented than that of global literature, the related research perspectives and contents were relatively diverse. For example, Zhu et al. (20) analyzed the interventions of TCMDPs in constructing the new rural cooperative medical care system in various historical stages in China with the help of historical comparison methods and summed up some realistic factors that may affect the efficiency of the system. Wang et al. (21) combed and analyzed nearly 30 TCMDPs issued since the 18th National Congress of the Communist Party of China in 2012 and introduced in detail the historical opportunities these policies brought to the future TCM development. Wang et al. (22), based on the content analysis methods, studied the TCMDPs involving the appraisal of senior professional titles of health workers and revealed the differences of TCMDPs among provinces and the problems related to the determination of indicators and participants. At the same time, some researchers, with the help of the multiple streams framework, explained what factors influenced the formation of the TCM Law and of which the specific policy goal and policy content (23). So far, it is not difficult to see that, although the above studies give us relatively multi-dimensional and comprehensive understandings of TCMDPs, they almost used the qualitative content analysis method to analyze specific TDMDPs. To our pleasure, some studies have also attempted to use quantitative textual methods to analyze some TDMDPs in recent years. The main research results mainly involve specific TCM study fields such as comprehensive development (24), inheritance and innovation (25), health service (26, 27), and preventive treatment of disease (28), which provide valuable insights into our better understanding of TCMDPs. However, these quantitative textual studies still had unsatisfying shortcomings, of which the apparent aspects, including single perspective and homogeneous method, only involved the theory of policy tools and had not used other quantitative textual techniques, which therefore made the study conclusions similar and little marginal contribution in giving us a panoramic response to TCMDPs evaluation.

### Applications of the PMC index model

Policy design and evaluation are complex and systematic projects that are vital links in the policy life cycle and relate to realizing policy goals (29–31). Hence, selecting appropriate evaluation tools or models has become an important research topic in policy evaluation (32). Only by choosing proper and scientific evaluation tools or models can the reliability of evaluation results be guaranteed (33). The PMC index model is a method to construct a unified evaluation indicator system based on the elements of the policy text and to evaluate a single or a series of policies quantitatively (34). Compared with other policy evaluation methods, the PMC index model has quantitative advantages and is widely used in many fields of policy evaluation. Specifically, Kuang et al. (35) evaluated eight cultivated land protection policies issued by the Chinese central government since 2004 and found great room for improvement regarding the combination of policy tools and content integrity. Dai et al. (36), based on a study on 16 green development policies concerning the Yangtze River Economic Belt, suggested five indicators, including policy timeliness, social benefits, audience scope, incentives, and constraints, significantly affected the quality of policy design. Liu and Liu (37) analyzed waste separation management policies issued by central government departments and provincial governments in the Yangtze River Delta from 2013 to 2021 and found that existing policies have great potential for optimization in terms of stricter constraint regulations, context-appropriate incentives, and the cultivation of market participants. In addition, many studies were using the PMC index model for fields, including new energy industry policy (38, 39), plastic bag ban policy (40), and textile industry policy (41), as well as ecological compensation policy (42), which reflected that this evaluation tool has good applicability and effectiveness in facilitating the quantitative policy studies and opening the black box in policy design. It is worth mentioning that some scholars have tried to combine emerging methods such as big-data text mining and the Latent Dirichlet Allocation (LDA) topic model to assist in identifying and measuring policy text content based on the PMC index model, which was a fruitful attempt to minimize subjectivity and instability in the policy evaluation process (43, 44). The applications of the PMC index model in previous studies are in the ascendant and have given us many gratifying research results. However, to our knowledge, no attention has been paid to applying this method in evaluating TCMDPs directly.

In conclusion, we found that previous studies still had some deficiencies in the field of TCMDP evaluation, which mainly includes the following two aspects: (1) The inherent components of TCMDPs had not been explored from the structural dimension of policy design. (2) Previous literature that lacked historical perspectives had neglected to take more TCMDPs with a long history into analyzing and only considered a few TCMDPs. The two made our overall and specific understanding of TCMDPs still been lacked. Therefore, it is necessary to comprehensively evaluate the current TCMDPs with the help of practical tools to provide improvement implications that can be used for reference in subsequent TCMDPs making. Through our review of the relevant literature about the PMC index model, we believe that it provides us with not only a new perspective for thinking but also a more operable, objective, and appropriate tool for assessing the strengths and weaknesses of TCMDPs. Hence, to narrow this academic gap, this study focuses on evaluating the consistency of TCMDPs in a more extended period and with a more extensive sample set, using the PMC index model and comprehensive policy evaluation indicator system to illustrate the advantages and disadvantages of TCMDPs and aiming to extend knowledge regarding the essential elements of high-quality policy texts.

## Materials and methods

### Data collection

The retrieval and acquisition of policy texts mainly come from the official portal websites of the Chinese central government and its ministries and commissions, the Beijing University Laws and Regulations Database (https://www.pkulaw.com/), CNKI (China National Knowledge Infrastructure) governmental policy texts database (http://r.cnki.net/kns/brief/result.aspx?dbprefix=gwkt), and other common search platforms (e.g., Google, Baidu, and Bing et al.) as a comparison and supplement for data collection. Besides, it should be pointed out that, to avoid the problems of repeated, invalid, and omitted collection of policy texts and to improve the effectiveness, comprehensiveness, and applicability of data, this study adopts the following retrieval strategies: (1) The main goals of policy texts needed to involve the field concerning TCM development, and crucial expressions/words (e.g., “TCM undertakings,” “TCM development” et al.) should appear many times in the content of the policy text; (2) The period for TCMDPs retrieval was set to be from 1980 to 2022; (3) The issuing agencies of TCMDPs were at the national level, including the State Council, national ministries and commissions, their subordinate national bureaus, and other entities; (4) Policy texts such as plans, outlines, opinions, measures and notices that could fully characterize and interpret the relevant content of TCM development are mainly selected, while policy texts such as letters, approvals and other content that were too brief and mainly concerning about list publication, review results, project establishment or technical guidelines were eliminated. Thus, we obtained a total of 454 TCMDPs at the first stage. However, to further ensure the representativeness of the TCMDPs and the consistency in the evaluation process, this paper, based on conducting a secondary screening, excluded some policy texts that had been repealed (or revised) or only concerned with single-dimensional content for the TCM development. In the end, 121 policy texts that met the research requirements and were closely related to the research topic were carefully determined (some selected TCMDPs are shown in Table 1).

**Table 1:** The selected TCMDPs in this study.

**Code**	**Policy name**	**Issuing agency**	**Date issued**
P5	Notice on Strengthening the Construction of Specialty of TCM	MH	Sep 26, 1983
P6	Several Opinions on the Development of TCM	SC	Jan 4, 1986
P15	Opinions on Effectively Strengthening the Work of TCM in Rural Areas	MH, NATCM	Apr 2, 1999
P17	10th Five-Year Plan for TCM	NATCM	Sep 04, 2001
P20	Guiding Opinions on Strengthening International Scientific and Technological Cooperation in TCM	NATCM	Jun 3, 2002
P39	Outline for the Development of Scientific Research in TCM (2006–2020)	NATCM	Sep 26, 2007
P49	Several Opinions on Supporting and Promoting the Development of TCM	SC	Apr 21, 2009
P52	Notice on Issuing Guidelines for the Construction of TCM Culture in TCM Hospitals	NATCM	Aug 4, 2009
P53	Notice on Strengthening the Staffing of TCM Hospitals by Leveraging the Advantages of TCM Characteristics	NATCM	Aug 7, 2009
P55	Notice on Issuing Guiding Opinions on Strengthening the Construction of Key Disciplines of TCM	NATCM	Oct 20, 2009
P70	Several Opinions on Promoting the Development of TCM Service Trade	MC, MFA, ME, etc.	Mar 5, 2012
P87	Opinions on Strengthening Supervision and Management of TCM	NHFPC, NATCM	Feb 5, 2016
P91	The 13th Five-Year Plan for the Information Development of TCM	NATCM	Nov 30, 2016
P94	Several Opinions on Accelerating the Construction of Science and Technology Innovation System of TCM	NATCM	Dec 22, 2016
P104	Notice on Carrying out the Pilot Work of Clinical Collaboration between TCM and Western Medicine for Major and Difficult Diseases	NATCM, NHFPC, HBLSD	Feb 28, 2018
P105	Measures for the Administration of the Construction of the National Comprehensive Reform Pilot Zone of TCM	NATCM	Jul 25, 2018
P109	Opinions on Promoting the Inheritance, Innovation and Development of TCM	CCCPC, SC	Oct 20, 2019
P114	Notice on Several Policies and Measures for Accelerating the Characteristic Development of TCM	SC	Jan 22, 2021
P120	Notice on Issuing the Development Plan for Promoting the High-quality Integration of TCM into the Belt and Road Initiative (2021–2025)	NATCM, LGPCBR	Dec 31, 2021

TCM: Traditional Chinese Medicine; CCCPC: Central Committee of the Communist Party of China; SC: State Council; MH: Ministry of Health (It changed into National Health and Family Planning Commission in 2013 and National Health Commission in 2018); NATCM: National Administration of Traditional Chinese Medicine; MC: Ministry of Commerce; MFA: Ministry of Foreign Affairs; ME: Ministry of Education; MST: Ministry of Science and Technology; MF: Ministry of Finance; MC: Ministry of Culture (It changed into Ministry of Culture and Tourism in 2018); GAC: General Administration of Customs; STA: State Taxation Administration; GAQSIQ: General Administration of Quality Supervision, Inspection and Quarantine (It changed into State Administration for Market Regulation in 2018); NFA: National Forestry Administration (It changed into National Forestry and Grassland Administration in 2018); CNIPO: China National Intellectual Property Administration, SAFE: State Administration of Foreign Exchange, NHFPC: National Health and Family Planning Commission (It changed into National Health Commission in 2018); HBLSD: Health Bureau of the Logistics Support Department of the CPC Central Military Commission; LGPCBR: the Leading Group for Promoting the Construction of the Belt and Road. P70'S issuing agencies included MC, MFA, ME, MST, MF, MC, MH, GAC, STA, GAQSIQ, NFA, CNIPO, NATCM, and SAFE.

### Methods

#### Steps to build the PMC index model

**Figure 1:**
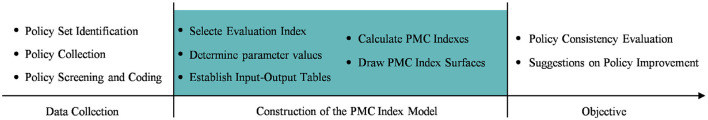
Construction framework for the PMC index model.

Referring to the research results of Ruiz Estrada (34), we comprehensively considered the different characteristics of each dimension of TCMDPs and then constructed the PMC index model and the relevant evaluation indicator system and carried out a quantitative analysis, which mainly consisted of four steps: (1) the pre-treatment (e.g., filtration and coding et al.) of the TCMDPs obtained; (2) the construction of a relevant evaluation index system for further quantitative analysis; (3) the classification of variables and determination of parameters, (4) the construction of a multi-input-output table; (5) the measurement of the PMC Index; and (6) the plotting of the PMC-Surface (Figure 1).

#### Construction of the PMC index model

The PMC evaluation index system for TCMDPs was created based on a combination of the classical framework of the PMC index model and the interpretation according to the specific contents of TCMDPs. The evaluation system was composed of 10 first-level indicators, namely, policy structure, policy tendency, policy orientation, policy timeliness, policy area, policy perspective, guarantees and incentives, object included, and issuing agency, which were further subdivided into 40 second-level indicators that covered the information and structural elements of TCMDPs as much as possible. The selection of evaluation indicators and their related explanations and references are shown in Table 2.

**Table 2:** Structure of the evaluation indicators system and criteria for the second-level index.

**First-level indicator**	**Code**	**Second-level indicator**	**Code**	**Evaluation criteria**	**References**
Policy structure	X1	Reliable basis	X1-1	Determine whether the policy involves a reliable basis, clear objective, scientific program, or detailed planning: if it does, the value is 1; if not, the value is 0.	(43)
		Clear objective	X1-2		
		Scientific program	X1-3		
		Detailed planning	X1-4		
Policy tendency	X2	Supervision	X2-1	Determine whether the policy involves administrative supervision, suggestion, description, guidance, or prediction: if it does, the value is 1; if not, the value is 0.	(45)
		Suggestion	X2-2		
		Description	X2-3		
		Guidance	X2-4		
		Prediction	X2-5		
Policy orientation	X3	Input-based	X3-1	Determine whether the policy orientation is input-based, process-based, or output-based: if it does, the value is 1; if not, the value is 0.	(46–48)
		Process-based	X3-2		
		Output-based	X3-3		
Policy timeliness	X4	Long-term	X4-1	Determine whether the impactful period of the policy is over the long term (≥6 years), the medium term (2–5 years), or the short term (< 1 year) period: if it does, the value is 1; if not, the value is 0.	(39)
		Medium-term	X4-2		
		Short-term	X4-3		
Policy area	X5	Talent training	X5-1	Determine whether the content of the policy involves talent training, science and technology, TCM industry, Integration of TCM with western medicine, service capability, cultural inheritance, facility construction, institutional system, or external exchange: if it does, the value is 1; if not, the value is 0.	(21)
		Science and technology	X5-2		
		TCM industry	X5-3		
		Integration of TCM with western medicine	X5-4		
		Service capability	X5-5		
		Cultural inheritance	X5-6		
		Facility construction	X5-7		
		Institutional system	X5-8		
		External exchange	X5-9		
Policy perspective	X6	Macro-level	X6-1	Determine whether the policy is enacted and implemented at the Macro-level, the Meso-level, or the Micro-level: if it is, the value is 1; if not, the value is 0.	(37)
		Meso-level	X6-2		
		Micro-level	X6-3		
Guarantees and incentives	X7	Organization	X7-1	Determine whether the policy involves organization, financial support, mobilization, or assessment: if it does, the value is 1; if not, the value is 0.	(35)
		Financial support	X7-2		
		Mobilization	X7-3		
		Assessment	X7-4		
Object included	X8	Administration	X8-1	Determine whether the object of the policy includes administration, healthcare institutions, or other social subjects: if it does, the value is 1; if not, the value is 0.	(36)
		Healthcare institution	X8-2		
		Social force	X8-3		
Issuing agency	X9	The State Council	X9-1	Determine the administrative level of the issuing agency of the policy: when the issuing agency is/are the State Council, multiple ministerial agencies, single ministerial agency, and lower, single ministerial agency, multiple deputy ministerial agencies, or single deputy ministerial agency, the value is 1, 0.9, 0.8, 0.7, 0.6, or 0.5, respectively.	(44)
		Multiple ministerial agencies	X9-2		
		Single ministerial agency and lower	X9-3		
		Single ministerial agency	X9-4		
		Multiple deputy ministerial agencies	X9-5		
		Single deputy ministerial agency	X9-6		

#### Measurement of the PMC index

The second-level indicators under the fore eight first-level indicators all obey the [0, 1] binary distribution shown in Equation (1), i.e., if the contents of the policy text satisfy the specific judgment conditions, the value of the corresponding second-level indicator is 1; otherwise, it is 0. Moreover, since the second-level indicators under X9 are mutually exclusive in classification, a separate provision has been made for parameter assignments to facilitate the scoring of the indicators, i.e., the values of second-level indicators under X9 are assigned in a decreasing way using Equation (2). All second-level indicators have the same importance or weights in the input-output table. The first-level indicators were calculated by summarizing all the second-level indicators, and the final PMC index was obtained by summing up the values of all variables using Equation (3). Finally, the surface graph, to display the resulting PMC index matrix more intuitively, was drawn based on Equation (4).

The results of the PMC index calculated by Equation (3) can be used to evaluate the consistency of TCMDPs. Since the design of our evaluation indicator system included nine first-level indicators, the theoretical values of the calculated PMC index should be in the [0, 9] interval. Referring to the practice of previous literature (37, 45) and combining the research needs of this study, we classify the measured PMC index values into six levels of consistency. Specifically, the values of the PMC index belong to the [0, 4) interval, [4, 6) interval, [6, 7) interval, [7, 8) interval, [8, 9) interval, or equal to nine, indicating that TCMDPs have “poor performance,” “acceptable performance,” “good performance,” “excellent performance,” “superb performance,” and “perfect performance,” respectively (Table 3).


(1) $$\begin{array}{l} X_{t-k_{t}}\sim B\left[0,1\right]\;\&\;X_{t-k_{t}}=\left\{PR:\left[0,1\right]\right\},t=1,2,\cdots \;,8 \end{array}$$

(2) $$\begin{array}{l} X_{9-k_{9}}=1.1-0.1\times k_{9},k_{9}=1,2,\cdots \;,6 \end{array}$$

(3) $$\begin{array}{l} PMC=\left[\begin{array}{c} X_{1}\left(\sum_{j=1}^{4}\frac{X_{1j}}{4}\right)+X_{2}\left(\sum_{j=1}^{5}\frac{X_{2j}}{5}\right)+X_{3}\left(\sum_{j=1}^{3}\frac{X_{3j}}{3}\right)+\\ X_{4}\left(\sum_{j=1}^{3}\frac{X_{4j}}{3}\right)+X_{5}\left(\sum_{j=1}^{9}\frac{X_{5j}}{9}\right)+X_{6}\left(\sum_{j=1}^{3}\frac{X_{6j}}{3}\right)+\\ X_{7}\left(\sum_{j=1}^{4}\frac{X_{7j}}{4}\right)+X_{8}\left(\sum_{j=1}^{3}\frac{X_{8j}}{3}\right)+X_{9}\left(1.1-0.1k_{9}\right) \end{array}\right] \end{array}$$

(4) $$\begin{array}{l} PMC\text{\_}Surface=\left(\begin{array}{c} X_{1}\;X_{2}\;X_{3}\\ X_{4}\;X_{5}\;X_{6}\\ X_{7}\;X_{8}\;X_{9} \end{array}\right) \end{array}$$

**Table 3:** Consistency categories for TCMDPs.

**PMC index interval**	**[0, 4)**	**[4, 6)**	**[6, 7)**	**[7, 8)**	**[8,9)**	**9**
Policy consistency	Poor	Acceptable	Good	Excellent	Superb	Perfect
Grade code	E	D	C	B	A	A+

## Results and discussion

### Analysis of the inter-annual change in the number of TCMDPs issued

**Figure 2:**
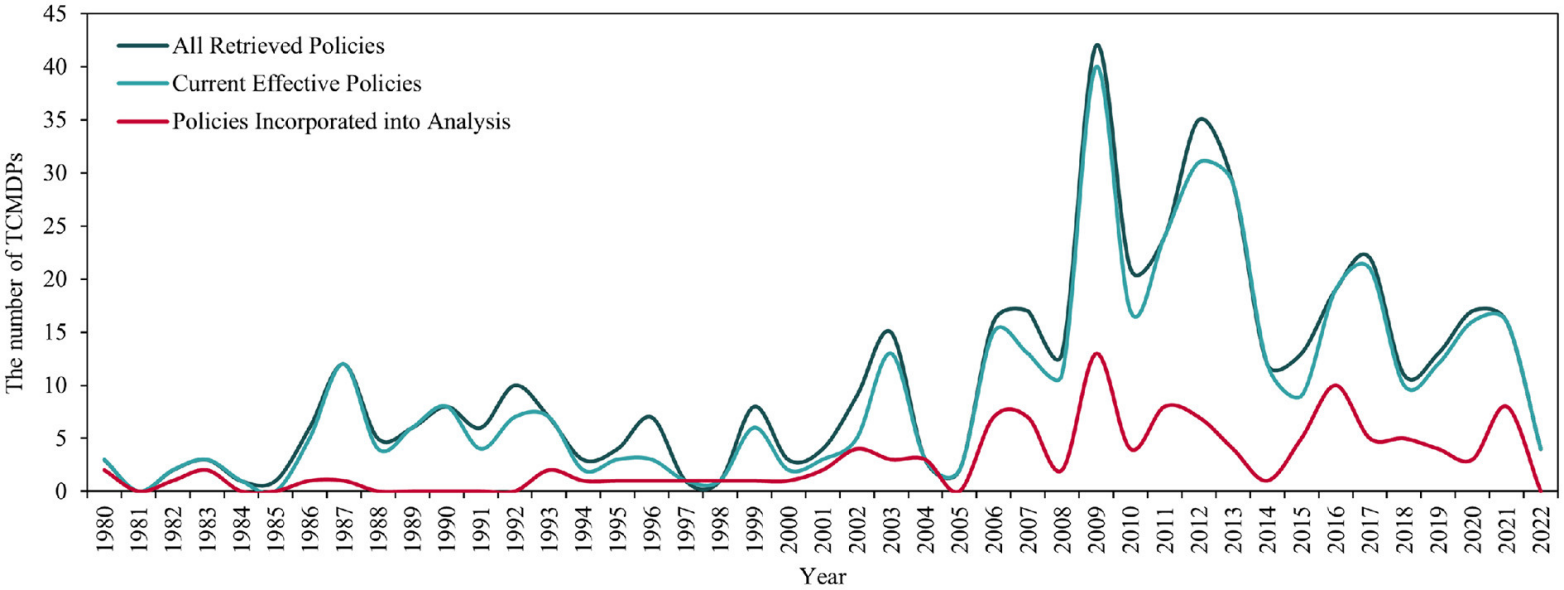
Inter-annual variation curve of the number of TCMDPs issued.

According to the inter-annual changes in the number of TCMDPs issued in Figure 2, we chose 2003 and 2011 as two particular time points to divide the time axis into three stages for facilitating analysis and discussion. In the first stage (1980–2002), the inter-annual number of TCMDPs issued remained low and did not change sharply. Instead, the number of TCMDPs issued had significantly increased since the Chinese central government established the NATCM, which indicated the government had a greater interest in developing TCM. Generally, this stage is a foundational period for the formation of TCMDPs and institutional mechanisms since the reform and opening up in China, in which the central government has accordingly issued a series of basic TCMDPs such as “Opinions on Strengthening Higher Education of TCM,” “National Regulations on the Work of TCM Hospitals” and “Several Opinions of the State Council on the Development of TCM” for providing essential solid support for the TCM development in the early stage of economic system reform. In the second stage (2003–2010), the inter-annual changes in the number of TCMDPs issued were dramatic. In April 2003, the State Council of PRC (People's Republic of China) issued the “Regulations of the People's Republic of China on TCM,” which motivated relative departments to pay more attention to TCMDPs design and made the number of TCMDPs issued quickly reach a small peak that year. In March 2009, CCCPC (Central Committee of the Communist Party of China) and SC (State Council of PRC) promulgated the “Opinions on Deepening the Reform of the Medical and Health System” and launched a new round of reform of the medical and health system, which pointed out the need to play the role of TCM further, and jointly promoted the intensive formation of many subsequent TCMDPs (e.g., “Several Opinions on Supporting and Promoting the Development of TCM,” “Opinions on Further Playing the Role of TCM in Deepening the Reform of the Medical and Health System” and “Implementation Opinions on Simultaneously Promoting the Comprehensive Reform of Public TCM Hospitals,” et al.). In the third stage (from 2012 to the present), the number of TCMDPs issued gradually declined. The Chinese central government has promulgated some policies, including “Outline of TCM Development Strategic Planning (2016–2030)” and “Opinions on Promoting TCM Inheritance, Innovation and Development” et al., which promoted TCM undertakings enter a high-quality and innovative period for better adapting to the development requirements and meeting the public needs in the new era. In conclusion, judging from the overall timeline and the content of specific policy issued, we suggested that the evolutionary trend of TCMDPs follow the path: “generation of the embryonic framework—system expansion—system refinement and improvement.”

### Holistic evaluation of TCMDPs

**Figure 3:**
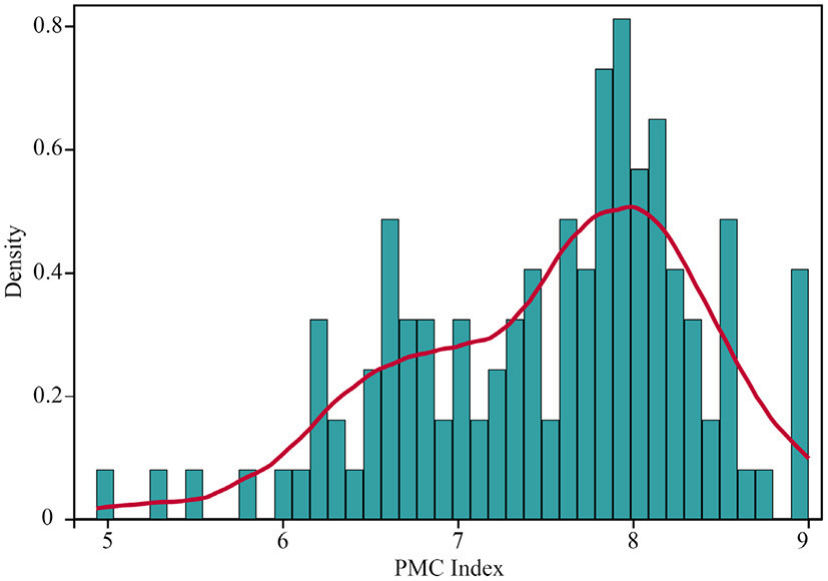
The kernel density curve of PMC index values of 121 TCMDPs. Bin = 40, start = 4.933, bandwidth = 0.102; the kernel density was estimated by the Epanechnikov method.

The distribution of PMC index values of the 121 TCMDPs included in the study is shown in Figure 3. 121 TCMDPs' total mean values of the PMC index were 7.530 ± 0.835, of which 70 (57.85%) TCMDPs' PMC index values were over that. Specifically, in our analysis, the number of TCMDPs belonging to perfect grade, superb grade, excellent grade, good grade, and acceptable grade was 4 (3.31%), 35 (28.93%), 50 (41.32%), 28 (23.14%), and 4 (3.31%), respectively. At the same time, we did not find any TCMDPs belonging to a poor grade in the evaluation process. From the kernel density curve in Figure 3, it can be seen that the distribution of PMC index values shows an apparent left-biased shape (Skewness = −0.592, Median = 7.722), indicating that the overall quality of TCMDPs is nearly at an excellent level, while some TCMDPs show low-quality performance simultaneously needing further targeted improvement.

**Figure 4:**
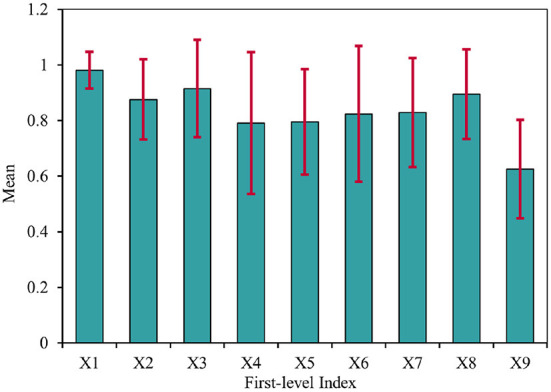
The mean values of nine first-level indicators.

Figure 4 provides the statistical results of each first-level indicator. The mean values of policy structure (X1), policy tendency (X2), and policy orientation (X3) reached 0.981 ± 0.066, 0.876 ± 0.144, and 0.915 ± 0.175, respectively, which indicated that TCMDPs generally had the completeness of the internal structure and the coordination of the elements, as well as the characteristics of comprehensive and precise content, simultaneously considered different policy tendency (e.g., supervision, suggestion, description, guidance, and prediction), and could follow the “input-process-output” policy chain. The mean value of policy timeliness (X4) is 0.791 ± 0.255, suggesting that TCMDPs could balance the long-term and short-term policy effects for maintaining the stability of the development of the TCM undertakings. The mean value of policy area (X5) is 0.795 ± 0.190, confirming the diversity and difference of goals between TCMDPs, and showing that it is necessary to make concerted efforts from various fields such as talents education, technological innovation, cultural inheritance, and system construction to realize the double-spiral development of soft power and hard power of TCM undertakings. Due to the promulgators of the TCMDPs included in this study being all at the national level, many TCMDPs directly made administrative instructions for regions (including provinces, municipalities, and autonomous territories, et al.), grassroots (including districts, counties, and towns et al.) and specific units. The policy perspective of most TCMDPs had macro, meso, and micro levels concurrently, so the mean value of policy perspective (X6) also reached 0.824 ± 0.244 correspondingly. The mean values of guarantees and incentives (X7) and object included (X8) were 0.829 ± 0.196 and 0.895 ± 0.161, respectively, indicating that, generally, TCMDPs can use various guarantees and incentives measures and incorporate multiple social subjects to promote the smooth and effective achievement of different policy goals. Additionally, it should be pointed out that due to the small number of documents issued by the State Council (including its General Office), most TCMDPs were issued by NATCM or other national ministries, resulting in the mean value of the issuing agency (X9) being only 0.625 ± 0.177, which is only slightly higher than the minimum value we prescribed of 0.5.

### Specific evaluation of TCMDPs

**Figure 5:**
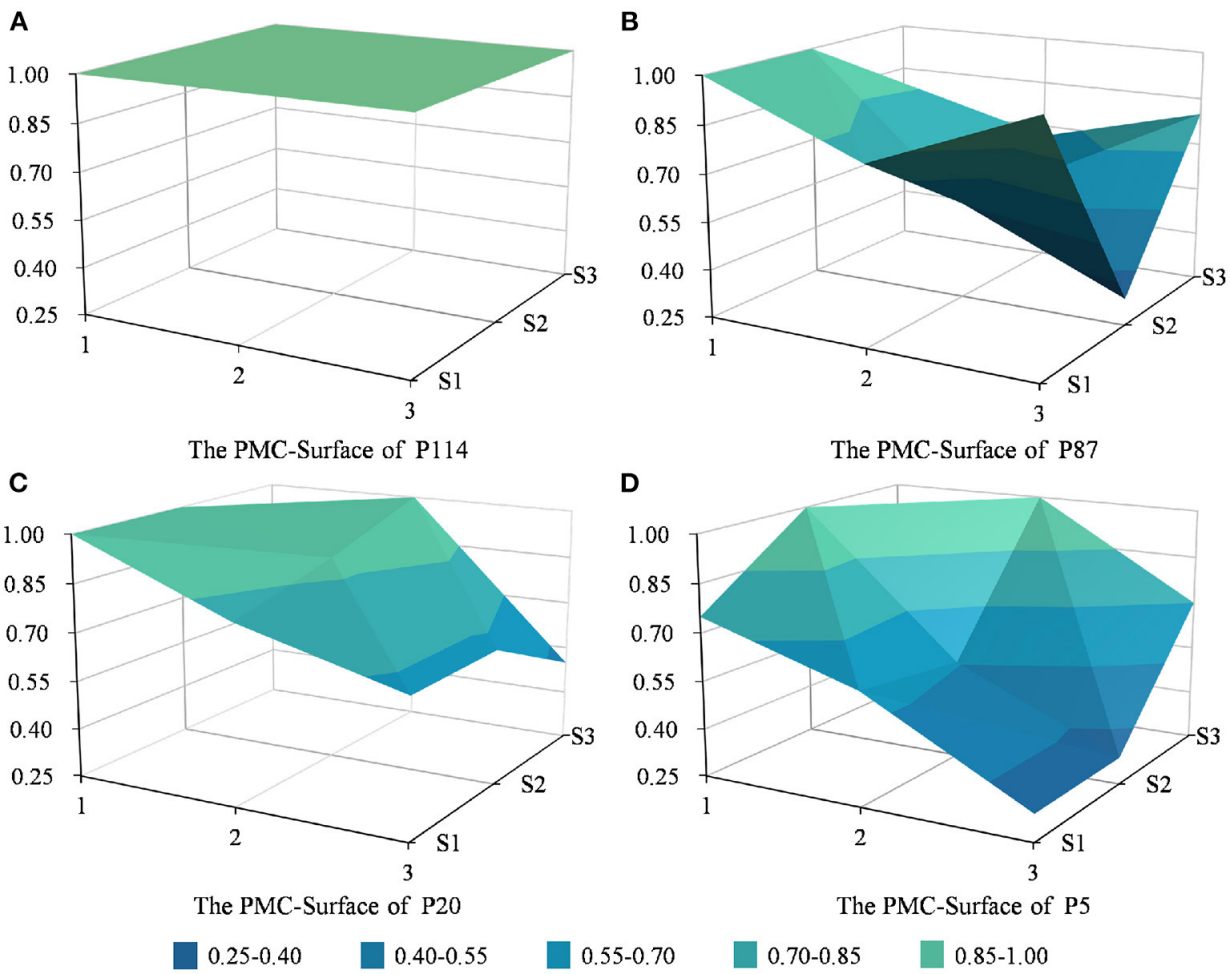
PMC surface charts of P114, P87, P20, and P5.

Applying the evaluation indicator system and PMC index model, we calculated and confirmed the multi-input/output matrix for the 19 TCMDPs, as shown in Supplementary Table S1, as well as the values of the first-level indicator for each policy and the corresponding PMC index, as shown in Table 4. Then, we also used the initial score table of the second-level evaluation indicators and the plots of PMC surfaces, combined the specific TCMDPs to focus on analyzing the consistencies of TCMDPs to make better micro-comparison between various TCMDPs and identify the features of those with multiple perspectives (Table 5 and Figure 5).

**Table 4:** The PMC index values and grades of selected TCMDPs.

**Policy**	**X1**	**X2**	**X3**	**X4**	**X5**	**X6**	**X7**	**X8**	**X9**	**PMC**	**Grade**	**Rank**
P5	0.750	0.600	0.333	1.000	0.556	0.333	0.250	1.000	0.700	5.522	D	19
P6	0.750	0.800	1.000	1.000	0.556	0.333	0.750	0.667	1.000	6.856	C	14
P15	1.000	1.000	1.000	1.000	0.889	1.000	1.000	1.000	0.800	8.689	A	3
P17	1.000	1.000	1.000	0.667	0.889	1.000	0.750	1.000	0.500	7.806	B	9
P20	1.000	0.800	0.667	1.000	0.889	0.667	0.750	1.000	0.500	7.272	B	11
P39	0.750	1.000	0.667	1.000	0.889	0.333	0.500	0.667	0.500	6.306	C	17
P49	1.000	0.800	1.000	1.000	1.000	1.000	0.750	1.000	1.000	8.550	A	4
P52	1.000	0.800	0.667	1.000	0.778	0.333	0.500	1.000	0.500	6.578	C	16
P53	1.000	0.800	0.333	1.000	0.333	0.667	0.500	0.667	0.500	5.800	D	18
P55	1.000	0.800	1.000	1.000	0.778	1.000	0.750	1.000	0.500	7.828	B	8
P70	1.000	0.800	1.000	1.000	1.000	0.667	0.750	1.000	0.900	8.117	A	5
P87	1.000	0.800	1.000	1.000	0.556	0.333	0.750	0.667	0.800	6.906	C	13
P91	1.000	1.000	1.000	0.667	0.889	1.000	0.750	1.000	0.500	7.806	B	9
P94	1.000	1.000	1.000	1.000	0.889	0.667	1.000	1.000	0.500	8.056	A	6
P104	1.000	0.800	1.000	0.667	0.667	0.333	1.000	1.000	0.800	7.267	B	12
P105	1.000	0.800	1.000	0.667	0.333	0.333	1.000	1.000	0.500	6.633	C	15
P109	1.000	1.000	1.000	1.000	1.000	1.000	1.000	1.000	1.000	9.000	A+	1
P114	1.000	1.000	1.000	1.000	1.000	1.000	1.000	1.000	1.000	9.000	A+	1
P120	1.000	1.000	1.000	0.667	1.000	0.667	1.000	1.000	0.600	7.933	B	7

**Table 5:** Mean values of second-level indicators in different dimensions.

**Indicator**		**Performance**	**Mean**	**Within SD**	**Pearson Chi-2/fisher's exact**	***p*-value**
X1	X1-1	Excellent	0.950	0.218	–	0.008
	X1-2	Excellent	1.000	0.000		
	X1-3	Excellent	1.000	0.000		
	X1-4	Excellent	0.975	0.156		
X2	X2-1	Excellent	0.909	0.289	142.917	< 0.001
	X2-2	Excellent	0.934	0.250		
	X2-3	Excellent	1.000	0.000		
	X2-4	Excellent	0.975	0.156		
	X2-5	Acceptable	0.562	0.498		
X3	X3-1	Good	0.810	0.394	27.370	< 0.001
	X3-2	Excellent	0.942	0.234		
	X3-3	Excellent	0.992	0.091		
X4	X4-1	Acceptable	0.545	0.500	76.920	< 0.001
	X4-2	Good	0.826	0.380		
	X4-3	Excellent	1.000	0.000		
X5	X5-1	Excellent	0.967	0.180	184.205	< 0.001
	X5-2	Excellent	0.967	0.180		
	X5-3	Acceptable	0.612	0.489		
	X5-4	Acceptable	0.669	0.472		
	X5-5	Excellent	0.917	0.276		
	X5-6	Acceptable	0.628	0.485		
	X5-7	Excellent	0.917	0.276		
	X5-8	Excellent	0.934	0.250		
	X5-9	Acceptable	0.545	0.500		
X6	X6-1	Excellent	0.917	0.276	15.062	< 0.001
	X6-2	Good	0.826	0.380		
	X6-3	Good	0.727	0.447		
X7	X7-1	Excellent	0.967	0.180	51.580	< 0.001
	X7-2	Good	0.777	0.418		
	X7-3	Acceptable	0.653	0.478		
	X7-4	Excellent	0.917	0.276		
X8	X8-1	Excellent	0.992	0.091	37.446	< 0.001
	X8-2	Excellent	0.934	0.250		
	X8-3	Good	0.760	0.429		
X9	X9-1	–	0.058	0.233	–	–
	X9-2		0.116	0.320		
	X9-3		0.132	0.339		
	X9-4		0.041	0.199		
	X9-5		0.017	0.127		
	X9-6		0.636	0.481		

SD, standard deviation. The mean values of the second-level indicators in different dimensions indicated the quality level of TCMDPs; Specifically, the mean values of the second-level indicators belonged to [0.9,1.0], [0.7,0.9), [0.5,0.7), [0.3,0.5), and [0.0,0.3), indicating that TCMDPs had “excellent performance,” “good performance,” “acceptable performance,” “non-satisfactory performance,” and “poor performance,” respectively. When the expected values in any contingency table cell were below 5, the Pearson Chi-square test was unsuitable for analysis, so we used Fisher's Exact Test, which does not allow us to obtain the specific statistic.

(1) Policy Structure (X1)

The mean values of second-level indicators, X1-1, X1-2, X1-3, and X1-4 of 121 TCMDPs included in this study were 0.950, 1, 1, and 0.975, respectively, which shows that most of TCMDPs, based on the existing political backgrounds (e.g., top-level policy designs, strategic layouts, meeting spirits et al.) and objective situations (e.g., significant issues, realistic challenges, social needs et al.), could set clear goals and form policy intentions for giving appropriate attention and responses to particular or global areas of TCM development. However, few policies ignore the description of the policy basis (e.g., P39) and the refinement of the policy content (e.g., P6).

(2) Policy Tendency (X2)

The shares of TCMDPs involving supervision, suggestion, guidance, and prediction in different policy tendencies were 90.91, 93.39, 97.52, and 56.20%, respectively. All TCMDPs had descriptive tendencies, and 50.41% of TCMDPs contained multiple policy tendencies simultaneously. Our study pointed out that, in the design process of TCMDPs, the policy tendencies, including supervision, suggestion, description, and guidance, have been generally paid attention to, but the prediction tendencies have been relatively weakened. In addition, some policies (e.g., P20) aimed at promoting international cooperation and exchanges in the field of TCM science and technology, thus weakening the role of supervision to a certain extent.

(3) Policy Orientation (X3)

The number of policy texts having input-based, process-based, and output-based orientations accounted for 80.99, 94.21, and 99.17%, respectively, and 78.51% of policy texts had the three at the same time, indicating that TCMDPs commonly concerned about the connection between selections of policy resources, the integration processes of policy elements, and the forms of policy results. In other words, current TCMDPs could strive to facilitate the smooth input of resources at the front end of the policy value chain, form a rational allocation of elements in the middle, and make overall estimates of policy results at the end, to effectively guide participants and all public sectors to establish reasonable expectations and make the achievement of the goals together. Meanwhile, some policies (e.g., P52) lacked attention to the input of resources, which might be detrimental to improving efficiency in policy implementation.

(4) Policy Timeliness (X4)

82.64 and 54.55% of the policy texts had medium-term and long-term policy impacts. All policy texts had short-term practical impacts joining medium-term or long-term impacts, while 17.36% had only short-term impacts. Combining with the review of specific policy texts, we found that TCMDPs with medium or long-term impact mostly belonged to certain policy types, such as guiding opinions, unique plans, and development outlines. For example, P70 proposed to “establish a complete TCM service trade management system in about 5 years”; P94 planned to build a high-quality TCM technology innovation system in an all-round way by 2030; P120 made an outlook on the goal and vision of more integration of TCM into the mainstream medical system of countries related to the “Belt and Road” in 2035.

(5) Policy Area (X5)

25.62% of the policy texts covered the nine major areas of TCM undertakings summarized in this study, and the share of policy texts involving five or more of the nine was up to 85.12%. Besides, the number of policy texts focusing on talent training, scientific and technological research, service capability improvement, and facility construction accounted for 96.69, 96.69, 91.74, and 91.74%, respectively. In our analysis combining the measurement results with the actual situation, we suggested that the areas that TCMDPs focus on were prominent, where that had hindered the development of TCM for so many years and needed to maintain long-term policy follow-up. Instead, the outlier policy sample, P105, just cared about the service capabilities, infrastructure construction, and management system improvement in the TCM comprehensive reform pilot area, where the policy function was slightly monotonous.

(6) Policy Perspective (X6)

The shares of TCMDPs having macro-level, meso-level, and micro-level perspectives were 91.74, 82.64, and 72.73%, respectively, while the share of TCMDPs having all three perspectives was 61.16%, which shows that many TCMDPs at the national level could respond to practical matters and did good jobs in regional planning and grassroots guidance while implementing a global institutional arrangement. Simultaneously, a few TCMDPs only focused on a single-level policy area. For example, P87 only gave explanations from the macro-level construction of the TCM regulatory system; P104 only guided the pilot work of clinical collaboration between TCM and Western medicine from a micro-level perspective.

(7) Guarantees and Incentives (X7)

The most frequently used guarantees and incentives measures of TCMDPs were organization (X7-1, 96.69%), assessment (X7-4, 91.74%), and financial support (X7-2, 77.69%), as well as mobilization (X7-3, 65.29%) from top to down. 49.59% of TCMDPs concurrently used the above four measures, while only two TCMDPs used only one of the four, indicating that the realization of complex and interrelated TCM development goals needed to rely on the combination of relatively comprehensive measures. From the perspective of abnormal policy samples, for example, P5 only emphasized the requirement to form a specific organizational guarantee in strengthening the construction of TCM specialties while ignoring the connection and application of other measures.

(8) Object Included (X8)

92.56% of TCMDPs included administrations and healthcare institutions that might be impacted by policies, while 69.42% of TCMDPs included three types of objects simultaneously. On the one hand, it shows that the administrations and healthcare institutions played an important role in promoting TCM development. On the other hand, it tells us that TCM development should not rely solely on the internal forces of the administrations and healthcare institutions but should also introduce other social forces to form a wide-range and of multi-subject interactions. For example, P49 encouraged social forces to invest in the establishment of TCM preventive healthcare service institutions; P109 mobilized local governments to set up TCM development funds that embody the characteristics of government guidance, social capital participation, and market-oriented operation.

(9) Issuing Agency (X9)

63.64% of TCMDPs were issued independently by the NATCM and other departments at the deputy ministerial level and below, while the number of TCMDPs issued and implemented by ministries and commissions at the ministerial level was relatively small. It can be seen that, although NATCM played a significant role in promoting the TCM development and undertook a lot of policy formulation work, the participation of other national ministries and commissions in this aspect was not enough, which shows the difficulties in forming a cooperative relationship between governmental department at different administration level in the development of TCM. Meanwhile, although the number of TCMDPs directly issued by the State Council of PRC was only six, they all had top-level design nature and planning effects and could send a powerful policy signal to the development of TCM.

The PMC surface charts for selected policies were drawn based on the measurement results of the PMC index matrixes, enabling us to more intuitively see the advantages and disadvantages of TCMDPs in design according to the degree of the graphical concave and convex. According to the classification of relevant literature (34, 37), specifically, the values of the first-level indicators belong to the [0.9, 1.0] interval, [0.7, 0.9) interval, [0.5, 0.7) interval, [0.3, 0.5) interval, or [0.0, 0.3) interval, meaning that the corresponding indicators have “excellent performance,” “good performance,” “good performance,” “acceptable performance,” “non-satisfactory performance,” and “poor performance,” respectively.

As Figure 5 shows, PMC surface charts for P114, P87, P20, and P5 are located in a three-dimensional coordinate system, in which x-coordinates of the matrix are denoted 1, 2, and 3 in the figure, and the y-coordinates are denoted series 1, series 2, and series 3. In each PMC index surface, the convex part corresponds to a higher PMC index, and the concave portion corresponds to a lower PMC index. The value of each first-level indicator of P114 is one, so its surface shape is a plane. Due to the advantages and disadvantages of designs of each policy being reflected in different aspects except for something similar in policy structure (X1), policy tendency (X2), and policy timeliness (X4), the shapes of PMC index surfaces P87, P20, and P5 are pretty different. Specifically, the P87 performed better on policy tendency (X2) and policy orientation (X3) but languished on policy area (X5) and policy perspective (X6); the P20 performed better on policy area (X5) and object included (X8) but languished on issuing agency (X9). Moreover, the PMC surface chart of P5 showed a shape liking to the waterfall, sloping from the high-value side to the low-value side, due to its poor scores on policy orientation (X3), policy area (X5), and policy perspective (X6). In conclusion, compared with P20 and P5, policy texts with good consistency are exemplified by P114 and P87.

## Conclusions and implications

### Conclusions

The main goal of the current study, using the PMC index model, was to conduct a comprehensive evaluation of the design quality of 121 TCMDPs issued by the Chinese central government (including its various departments) over the past four decades and probe the level of consistency of TCMDPs from the holistic and specific perspectives, which let us obtain some interesting conclusions given below.

The holistic consistencies of TCMDPs selected in the current study had excellent and reasonable performances. Among the policy clusters studied, four policies were rated as “perfect,” 35 were rated as “superb,” 50 were rated as “excellent,” and 28 were rated as “good.” Only four were rated as “acceptable,” with the total mean values of the PMC index being 7.530 ± 0.835. In our view, most TCMDPs incorporated a wealth of structural components in the overall design and could sort out and present the collocation logic of multi-dimensional components. Whether it was from the PMC index absolute values or the distribution of the grade interval of the PMC index values, TCMDPs generally showed good consistency and the rationality of policy design. At the same time, only a tiny number of TCMDPs had outlier tendencies and abnormal characteristics in measurement values of the PMC index, first-level indicator, and second-level indicator. Specifically, the designs of TCMDPs had some points worthy of affirmation and our approval as follows: (The degree of internal coupling of the TCMDPs' policy system was relatively high. The policy structures (X1), tendencies (X2), and orientations (X3) could generally be taken into account as much as possible during the design process of TCMDPs. (2) The connection between policy timeliness (X4) and policy areas (X5) shown in the TCMDPs' internal components could form a policy impact layout combined time and space dimensions, balancing short-term and medium-long-term effects, as well as the micro and medium-macro fields in TCM developments. (3) TCMDPs thought over a variety of policy perspectives and guarantees and incentives measures and the appropriate connections between the two. (4) Due to the complexity and richness of the TCM development, unlike policies from other fields that affect only a single object, TCMDPs, to a certain, could be good at identifying the various related objects and guiding them to form sustainable cooperation and participation in achieving policy goals.

Besides, although most TCMDPs had excellent policy design and policy consistency, the potential weaknesses and inherent deficiencies in the design of TCMDPs also need our attention and analysis based on careful checks on the outlier policy samples. The deficiencies in the following aspects can be roughly summarized based on the previous PMC index measurement results and specific policy contents: (1) The few TCMDPs concentrated too much on the advancement of temporary work but failed to make reasonable assessments and clear explanations of policy goals and expected effects. (2) Partial TCMDPs had circumscribed focus on particular policy areas, emphasizing some essential topics, such as TCM industry, cultural inheritance, and external exchange. (3) Some policies unilaterally use administrative and mandatory measures such as strengthening the organization and regulations to promote the realization of policy goals, ignoring the application of economic and incentive measures such as financial support, publicity, and mobilization. (4) The governmental departments that issued TCMDPs were too centralized, i.e., most policies were promulgated by NATCM. In contrast, the number of policies promulgated by led or involved dominant national ministries or commissions were small, making it challenging to form sectoral cooperations at the national level to promote TCM improvement.

### Implications

Based on the above analysis, in our view, the findings of this study have significant implications for the understanding of how to optimize the structural design in TCMDPs design.

The combination and relationship of various elements in TCMDPs must be re-examined and optimized. TCMDPs are an essential carrier of the Chinese central government's will to support and encourage the sustainable and healthy development of TCM undertakings, of which transmissions of policy information (e.g., content composition, coverage, and impactful period et al.) are closely related to the actual policy effectiveness. Hence, some efforts should be made in time to achieve these goals, i.e., we should further re-examine and rationalize the existing policy elements and their corresponding relationships and start from the weaknesses to carry out targeted optimization. First, issuing agencies should follow the normative strategy in policy design, further improve the combination and relationship of different essential components of the policy, and form a complete policy structure framework of “base-target-path.” Second, issuing agencies should follow the scientific strategy in policy design, consider different policy tendencies, and appropriately enhance policy forecasting ability. Third, issuing agencies also should follow the holistic strategy in policy design, starting from the overall perspective of “time-space-process” and according to the actual needs for achieving policy goals, coordinate the content of policy timeliness, policy areas, and policy perspectives.

The double guarantee mechanisms and policy implementation measures need to be further strengthened. As mentioned above, the low PMC index values of some TCMDPs could be attributed to the lack of policy design in the dimension of safeguarding to a certain extent. However, TCM development is a complex systemic project that requires multi-departmental cooperation and multi-stakeholder participation. Hence, we emphasize that some work should be done as follows. On the one hand, several guarantee and incentive measures and frequency of financial support, as well as mobilization measures, will be further enhanced, providing an objective resource for the realization of TCM development goals and urging all parties to clarify their awareness of subject responsibility and to improve their willingness to act for the policy goals. On the other hand, it is necessary to clarify the specific responsibilities and potential participation possibilities of different national ministries and commissions in promoting TCM development and strengthen the synergy between NATCM and other ministries as well as commissions, i.e., according to different TCM development agendas, relevant departments can be involved in the design, promulgation, and implementation of TCMDPs in time, forming the functional integration of various administrative systems and providing more reliable and variable support for TCM development.

### Limitations and further works

We emphasize that the policy evaluation method used in this current study, the PMC index model is scarce in the TCMDPs. To a certain extent, this study can provide new ideas and lay a theoretical foundation for future policy evaluation of TCM development, which findings should help to improve our understanding of the design of TCMDPs.

However, this study also has some limitations due to time and scope constraints. First, the policy samples were selected in this study through rigorous and targeted screening, mainly including policies issued at the national level but not at the local level, which made the generalisability of these results subject to certain restrictions. Second, the PMC index model is a simple summation and averaging and affirms a priori that indicators from all aspects of the evaluation indicator system are equally important. Although the setting refinement and quantitative difference of second-level indicators may feedback the weights into the first-level indicators to a certain extent, it does not integrate information across each evaluation indicator, thereby making it less helpful and effective to identify the relationship and significant relative differences between the different evaluation indicators. Future research can be carried out as follows: (1) Pay more attention to the TCMDPs issued by local governments, which may have more characteristics based on local conditions of TCM development that need to be analyzed. (2) Combining AHP (Analytic Hierarchy Process) and other methods to assign weights to indicators at all levels to reflect the relative importance of each indicator may help us obtain a more objective understanding of policy consistency.

## Data availability statement

The original contributions presented in the study are included in the article/Supplementary material, further inquiries can be directed to the corresponding author.

## Author contributions

CY and SY wrote the draft of the manuscript and interpreted the results. CY, SY, YS, SA, YD, QZ, RW, YL, XH, and YW collected and analyzed the data. CY, JR, SA, YW, and XL interpreted partial results. CY, SY, ZM, and DC designed the study and provided administrative and material support. SY, YS, CJ, JR, and DC were involved in editing and revising each draft and provided overall guidance. ZM and DC provided administrative supports, comments, and suggestions in revisions of the paper. All authors approved the final submitted version.

## Conflict of interest

The authors declare that the research was conducted in the absence of any commercial or financial relationships that could be construed as a potential conflict of interest.

## Publisher's note

All claims expressed in this article are solely those of the authors and do not necessarily represent those of their affiliated organizations, or those of the publisher, the editors and the reviewers. Any product that may be evaluated in this article, or claim that may be made by its manufacturer, is not guaranteed or endorsed by the publisher.

## Abbreviations

TCM: traditional Chinese medicine
TCMDPs: traditional Chinese medicine development policies
PMC: policy modeling consistency
CCCPC: central committee of the communist party of China
SC: state council
MH: ministry of health (It changed into National Health and Family Planning Commission in 2013 and National Health Commission in 2018)
NATCM: national administration of traditional Chinese medicine
MC, ministry of commerce, MFA: ministry of foreign affairs
ME: ministry of education
MST: ministry of science and technology
MF, ministry of finance, MC: ministry of culture (It changed into Ministry of Culture and Tourism in 2018)
GAC: general administration of customs
STA: state taxation administration
GAQSIQ, general administration of quality supervision: inspection and quarantine (It changed into State Administration for Market Regulation in 2018)
NFA: national forestry administration (It changed into National Forestry and Grassland Administration in 2018)
CNIPO: China national intellectual property administration
SAFE: state administration of foreign exchange
NHFPC: national health and family planning commission (It changed into National Health Commission in 2018)
HBLSD: health bureau of the logistics support department of the CPC central military commission
LGPCBR: the leading group for promoting the construction of the belt and road
SD: standard deviation.
